# Blunted Heart Rate Response as a Potential Endophenotype of Substance Use Disorders: Evidence from High-Risk Youth

**DOI:** 10.3389/fped.2015.00066

**Published:** 2015-07-28

**Authors:** Brittany E. Evans, Kirstin Greaves-Lord, Anja S. Euser, Tess Koning, Joke H. M. Tulen, Ingmar H. A. Franken, Anja C. Huizink

**Affiliations:** ^1^Department of Child and Adolescent Psychiatry/Psychology, Erasmus University Medical Center, Sophia Children’s Hospital, Rotterdam, Netherlands; ^2^Department of Developmental Psychology, EMGO Institute for Health and Care Research, VU University Amsterdam, Amsterdam, Netherlands; ^3^Behavioral Science Institute, Radboud University, Nijmegen, Netherlands; ^4^Department of Psychology, Erasmus University Rotterdam, Rotterdam, Netherlands; ^5^Department of Psychiatry, Erasmus University Medical Center, Rotterdam, Netherlands

**Keywords:** heart rate, stress reactivity, substance use disorders, externalizing, familial risk

## Abstract

**Background:**

Children of parents with a substance use disorder (CPSUD) are at increased risk for developing problematic substance use later in life. Endophenotypes may help to clarify the mechanism behind this increased risk. However, substance use and externalizing symptoms may confound the relation between dysregulated physiological stress responding and familial risk for substance use disorders (SUDs).

**Methods:**

We examined whether heart rate (HR) responses differed between CPSUDs and controls. Participants (aged 11–20 years) were CPSUDs (*N* = 75) and controls (*N* = 363), semi-matched on the basis of sex, socioeconomic status, and ethnicity. HR was measured continuously during a psychosocial stress procedure. Substance use and externalizing symptoms were self-reported and mother-reported, respectively.

**Results:**

A piecewise, mixed-effects model was fit for HR across the stress procedure, with fixed effects for HR reactivity and HR recovery. CPSUDs showed a blunted HR recovery. CPSUDs reported drinking more frequently, were more likely to use tobacco daily, were more likely to report ever use of cannabis and used cannabis more frequently, and exhibited more externalizing symptoms. These variables did not confound the relation between familial risk for SUDs and a blunted HR recovery.

**Conclusion:**

Our findings suggest dysregulated autonomic nervous system (ANS) responding in CPSUDs and contribute to the accumulating evidence for ANS dysregulation as a potential endophenotype for SUDs.

## Introduction

Starting with adoption studies in the 1970s, several studies have demonstrated that the risk for developing substance use disorders (SUDs) is substantially higher in the children of parents with a substance use disorder [CPSUDs; e.g., Ref. (1–7)]. In genetically complex psychiatric disorders, such as SUDs, endophenotypes or biomarkers may help to explain risk for SUDs in CPSUDs. One potential endophenotype is dysregulation of the physiological response to stress.

Physiological stress responding can be indexed by the autonomic nervous system (ANS), which consists of the parasympathetic (PNS) and sympathetic nervous systems (SNS). In healthy individuals, the PNS maintains homeostasis and supports social engagement during rest (8, 9). Activation of the SNS and simultaneous deactivation of the PNS occur in response to a stressor, increasing heart rate (HR), and enabling the adaptive fight-or-flight response. Physiological recovery from the stressor entails deactivation of the SNS and activation of the PNS in order to return to homeostasis (9, 10).

Alterations in this normative adaptation of the ANS to stress have been theoretically linked to adverse outcomes. A low resting mean HR and reduced HR reactivity may indicate fearlessness, and thereby reduced inhibition to engaging in risky behavior (11). Similarly, low physiological reactivity could signify an inherent hypo-arousal, which leads individuals to actively seek out stimulation, sometimes in the form of risky behavior, in order to achieve a state of normalized physiological arousal (12–14). Thus, dysregulation of the ANS stress response could constitute an important underlying biological mechanism for risky behavior and in this way relay risk for developing SUDs.

Earlier studies have provided evidence for dysregulated physiological stress responding in patients with SUDs [e.g., Ref. (15)] as well as in CPSUDs (16–18). However, studies investigating specifically HR reactivity have been equivocal, indicating both *hypo*- and *hyper*-reactivity compared to controls (19–21). Thus, consensus is lacking at this point as to the nature of HR responses to stress in CPSUDs.

In sum, in the current study, we aimed to investigate whether HR reactivity differed in youth at high risk for developing SUDs (i.e., CPSUDs) compared to youth from the general population. In previous studies, it has been widely reported that CPSUDs have a higher tendency to engage in risky substance use and manifest higher symptomatology of externalizing problems (i.e., symptoms of attention-deficit hyperactivity, conduct and oppositional-defiant disorders) compared to those from the general population [e.g., Ref. (2, 5, 7, 22)]. Because both risky substance use (23) and externalizing problems (24) have been associated with blunted HR reactivity in youth, it is possible that the relation between familial risk for SUDs and dysregulated HR reactivity is confounded by substance use and/or externalizing symptomatology. Therefore, the second aim of this study was to examine whether any relation between risk group and HR reactivity was confounded by substance use (i.e., ever use of alcohol and cannabis, and daily tobacco use) and externalizing symptoms. The third aim of this study was to investigate whether *frequency* of substance use (alcohol and cannabis) confounded any relation between risk group and HR reactivity in a subset of youth for whom we had more detailed information on substance use. We tested confounding by first examining the relation between risk group and the potential confounder (i.e., ever use of alcohol and cannabis, daily tobacco use, externalizing symptoms, and in a subsample, frequency of alcohol and cannabis use). Second, we examined whether the potential confounder was related to HR reactivity. Third, we tested whether the relation between risk group and HR reactivity changed when controlling for the potential confounder.

## Materials and Methods

### Participants

#### Controls

Controls were part of a longitudinal general population study (25) in which participants were randomly drawn from registers of 35 municipalities in South Holland. During wave 2, 711 individuals (7–20 years) participated in a psychosocial stress procedure. Of these, data on HR were available for 636 individuals. To obtain a control group with the same range of ages as the CPSUD group (i.e., 11–20 years, see below), we excluded all control subjects younger than 11 years (*n* = 185). We furthermore excluded those with a parent who had been diagnosed with an SUD [based on the Composite International Diagnostic Interview (CIDI); (26); performed by a trained interviewer; *n* = 8]. We subsequently excluded 80 individuals in order to obtain a population semi-matched to the CPSUD group on the basis of sex, socioeconomic status (SES), and ethnicity. The matching procedure consisted of excluding individuals from the control group in groups of 10 in order to obtain a control group that did not differ significantly from the CPSUD group regarding sex, SES, and ethnicity. For example, if the CPSUD group had a significantly higher proportion of girls, we randomly selected 10 control boys and excluded them. This procedure led to a control sample of 363 subjects. No siblings were included in this sample. The mean age of youth in the control group was 15.50 (SD = 2.68) and 49.9% were boys. For a detailed description of the study sample (CPSUD and control samples) and design, please see Huizink et al. (27).

#### Children of Parents with a Substance Use Disorder

In order to examine familial risk for SUDs, we recruited a sample of CPSUDs as an addition to the general population sample. The CPSUD sample consisted of 75 youth (11–20 years) with at least one SUD-diagnosed parent [according to DSM-IV; (28)], mostly recruited via outpatient addiction care Bouman-GGZ clinics (South Holland), where their parents were in treatment (*n* = 69). Clinical staff provided parental SUD diagnoses. Study information was initially given by Bouman-GGZ clinical staff. Some participants (*n* = 6) were recruited by word of mouth as their parents were not in treatment. Parental SUD diagnoses were then obtained through a structured interview (CIDI) with the parent of all DSM-IV axis 1 disorders, performed by a trained interviewer. The mean age of youth in the CPSUD group was 15.87 years (SD = 2.42) and 53.3% were boys. Of the parents, 64.5% was in treatment for primarily alcohol use, 9.7% for primarily cannabis or sedatives use, and 25.8% for polydrug use or other drugs. Fathers made up 52.4% of the parents, mothers 44.4%, and both parents 3.2%. More than one child per family was allowed to participate in the study; the sample comprised 75 participants from 58 families; 31 were siblings. Non-independence of observations in this sample was assessed as a potential confounding factor (see Basic HR Response Model: Model 1 of Results).

For the third aim, we examined a subset of youth for whom detailed information was available on substance use. In the control sample, this included youth of 14 years and older (*n* = 243). This information was requested of all CPSUDs; however, in order to maintain balanced groups age-wise, we included only CPSUDs 14 years and older (*n* = 55).

### Procedure

**Figure 1:**
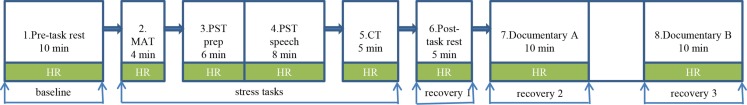
**Psychosocial stress procedure during which heart rate was measured continuously**. *HR, heart rate; MAT, mental arithmetic task; PST, public speaking task; prep, preparation; CT, computer task.

Participants were invited to a lab at the Erasmus University Rotterdam, the Erasmus University Medical Center, or at a location nearer to their homes. Upon arrival, an explanation of the psychosocial stress procedure was given, and participants completed questionnaires. Subsequently, the electrodes of the electrocardiogram (ECG) were attached and participants were told to breathe normally and to relax. After a 10-min pre-task rest period (baseline), the social stress tasks began, patterned after the well-validated Trier Social Stress Task, and designed to elicit a stress reaction (29–32). These entailed a mental arithmetic task, a public speaking task (preparation and speech), and a computer mathematics task (four stress task periods). The session ended with a 5-min resting period and a 25-min nature documentary, of which the first and last 10-min periods were used (recovery periods 1, 2, 3; see Figure 1). Written informed consent was obtained from all youth and their parents; youth received a gift certificate. The study was approved by the Medical Ethics Committee of the Erasmus University Medical Center.

### Measures

#### Externalizing Symptoms

Mothers of participants completed the Dutch version of the Child Behavior Checklist [CBCL; (33)]. Empirical findings have indicated that attention-deficit hyperactivity disorder, oppositional defiant disorder, and conduct disorder can be considered as a broad dimension of externalizing disorders (34). Therefore, to determine number of externalizing symptoms, scores on the three subscales pertaining to these disorders were summed.

#### Substance Use

We assessed substance use with a youth self-report Substance Use Questionnaire (SUQ). Variables were dichotomous for ever use of *alcohol* (use = at least one glass), *cannabis* (use = used at least once), and *tobacco* (no use = never smoked, smoked one or two cigarettes ever, currently smoked once in a while, or quit; daily use = smoked every day).

For the third aim, we utilized questions from an extended version of the SUQ. This version was only completed by youth from the control group who were 14 years and older, because of the expected low frequency of substance use in this sample. The extended version was completed by all youth from the CPSUD group; however, in order to maintain balanced groups age-wise, we only included CPSUD youth aged 14 and older in the analyses examining our third aim. *Frequency of alcohol use* consisted of the average number of alcoholic drinks usually consumed per week. *Frequency of cannabis use* pertained to the number of times cannabis was used per week, on average during the past 12 months. Data on frequency of tobacco use were not available.

#### Heart Rate

Heart rate was measured using a three-lead ECG, monitored constantly throughout the stress procedure. The ECG was sampled at 512 Hz and stored on a flashcard with a portable digital recorder (Vitaport™ System; TEMEC Instruments BV). Physiological data were imported and processed using a Vitascore™ software module (TEMEC Instruments BV) post-recording. A customized software program calculated the inter-beat intervals (IBI) of the ECG using R-top detection, resulting in IBI time series. This time series was inspected for detection and removal of artifacts. HR time series were calculated from these IBI time series, expressed in beats per minute, and averaged per period of the stress procedure. As an indicator of HR during the stress tasks, we used the maximum mean HR from any of the four stress task periods.

#### Covariates

Age, sex, body mass index (BMI), SES, and urbanicity were covariates. SES was based on the higher occupational level of either parent and coded into low, average, and high (35). Age and sex were self-reported. Height and weight were measured prior to the test session and used to calculate BMI. Urbanicity was based on the population rate [according to online national archives; (36)] of the home city/town of the participant at the time of the test session and coded into rural (<10,000 inhabitants), town (>10,000 inhabitants), and urban [>100,000 inhabitants; (37)].

### Statistical analysis

To confirm that the stressful tasks induced an increase in HR, we performed a manipulation check with a repeated measures analyses of variance (RM-ANOVA) in the entire sample (1 × 6) in IBM SPSS statistics version 20.

For the main analyses, a piecewise mixed-effects model was fit for HR across the stress procedure in statistical package R (38) using the “lmer” function from the lme4 package (39). Multilevel modeling is preferable in examining physiological stress measurements [e.g., Ref. (40)] because first, within-individual variation in mean HR across the procedure is controlled at the first level. If sufficient variance is observed in mean HR, this warrants the examination of between-individual predictors of these measurements. Moreover, this technique utilizes all data points, thereby efficiently handling missing data. Estimating piecewise models allows the examination of both reactivity and recovery in one parsimonious model.

The HR curve consisted of five measurement points, pertaining to the average HR of five periods: (1) baseline, (2) maximum HR during any of the four task periods, and (3–5) recovery periods 1, 2, and 3. We determined where to split the piecewise curve based on visual inspection [according to Ref. (41)]. The intercept was set at the first measurement in order to examine differences at the pre-task baseline period. At level 1, within-individual differences in HR measures across the stress procedure were controlled. At level 2, we added between-individual predictors of HR. We examined whether it was necessary to add a third level, controlling for family resemblance, as siblings were included in the CPSUD group. Between models, fit was compared using log likelihood ratio tests.

In order to examine whether CPSUDs and controls differed on HR reactivity, we first fitted models containing the intercept and slopes of HR measurements (basic HR model). Next, group (CPSUDs, controls) was added to the model. Both the main effect of group (effect of group on the intercept) and interactions with period (effect of group on slopes) were added.

In the case of a significant relation between group and HR reactivity, we then tested whether substance use and externalizing symptoms confounded this relation. We did this in three steps. First, we examined whether group was related to each potential confounder (i.e., ever use of alcohol and cannabis, no/daily smoking and externalizing symptoms) with a series of single-level logistic and linear regression models (“lm” and “lmer” functions, respectively). Second, we tested whether each potential confounder was related to HR reactivity by adding the potential confounder to the basic HR model as described above. Both main effects (effect of the potential confounder on the intercept) and interactions with period (effects of the potential confounder on slopes) were added. Third, we added each potential confounder, in separate models, to the model in which group was the main predictor of HR reactivity and examined whether the significant relation between group and HR reactivity remained. We performed this final step only for the potential confounders for which there was a significant relation in the first (relation between group and confounder) and second (relation between confounder and HR reactivity) steps.

We then examined our third aim of potential confounding effects of frequency of substance use on the relation between group and HR reactivity in a subsample of youth for whom we had more detailed information on substance use. We repeated the steps outlined in the previous paragraph, only this time with the potential confounders of frequency of alcohol use and frequency of cannabis use.

In all models, period was coded taking into account the time interval between measurements. All models were controlled for age, sex, BMI, SES, urbanicity, and interactions between these variables and period. All models were fit with maximum likelihood estimation, all continuous variables were centered, and categorical variables were coded beginning with *x* = 0. *P* values <0.05 were considered significant. Because *p* values are not estimated with the lmer function, we considered *T* values >2 or <−2 to be significant.

## Results

Descriptive statistics and group differences in the covariates are presented in Table 1.

**Table 1:** **Descriptive statistics for CPSUDs and controls**.

	Control		CPSUD		Group differences
	*N*	Mean (SD) or *F* (%)	*N*	Mean (SD) or *F* (%)	χ^2^ or *t*
Age	363	15.50 (2.68)	75	15.87 (2.42)	−1.12
Sex (boys; 0/girls; 1)	363	49.9/50.1	75	53.3/46.7	0.30
Body mass index	358	21.23 (3.69)	68	20.63 (3.45)	1.25
Ethnicity (Dutch; 0/non-Dutch; 1)	363	86.2/13.8	74	91.9/8.1	1.77
SES (low; 0/average; 1/high; 2)	361	5.8/62.3/31.9	67	1.5/76.1/22.4	5.39
Urbanicity (rural; 0/town; 1/urban; 2)	363	14.0/57.3/28.7	75	16.0/56.0/28.0	0.19

*CPSUD, children of parents with a substance use disorder; SES, socioeconomic status.

### Manipulation check

The RM-ANOVA showed that HR changed significantly across the procedure [*F*(3.32, 1386.90) = 382.62, *p* < 0.001, η^2^ = 0.48]; specifically, HR increased significantly, relative to the baseline period, during the mental arithmetic [*F*(1, 418) = 224.36, *p* < 0.001, η^2^ = 0.35], public speaking preparation [*F*(1, 418) = 86.95, *p* < 0.001, η^2^ = 0.17], public speaking speech [*F*(1, 418) = 303.41, *p* < 0.001, η^2^ = 0.42], and computer task [*F*(1, 418) = 31.56, *p* < 0.001, η^2^ = 0.07] periods. The stress procedure thus elicited the expected HR response.

### Basic HR response model: Model 1

We first examined the individual plots of HR across the stress procedure. In general and as expected, HR increased between baseline and the maximum HR during the tasks, and subsequently decreased from the maximum stress HR to recovery periods 1, 2, and 3. Therefore, we split the piecewise curve at maximum stress HR, yielding an HR reactivity slope (baseline, maximum stress) and an HR recovery slope (maximum stress, recovery 1, 2, and 3). Both of these slopes were included as fixed effects. We additionally included a random effect for the slope across the entire stress procedure (TestSlope) in order to control for within-individual variation in HR.

The empty model included only HR and TestSlope. We subsequently examined whether it was necessary to include a level controlling for family resemblance, as siblings were included in the CPSUD group. This model was not significantly improved above the empty model; therefore, for reasons of parsimony, we dropped this level, and fit a two-level model. Next, the covariates age, sex, BMI, SES, and urbanicity were included in the model. This was a significantly improved model above the empty model [χ^2^(21) = 794.27, *p* < 0.001]. The parameters for this basic HR response model are given in Table 2. Age was significantly related to the intercept (older youth had lower mean HR at the beginning of the stress procedure), HR reactivity, and HR recovery (older youth showed steeper HR reactivity and recovery slopes). Sex was significantly related to HR reactivity and recovery such that girls showed stronger reactivity and recovery. Model 1, the basic HR response model, thus included a random effect for HR across the entire procedure, fixed effects for the HR reactivity slope, the HR recovery slope, and all covariates, including main effects and interactions with both slope factors.

**Table 2:** **Parameter estimates for the basic HR model including covariates**.

*N* level 1	2044	
*N* level 2	412	

**Fixed effects**	**Estimate (SE)**	***t***

*Intercept*	**79.27 (4.10)**	**19.32**
Age	**−0.66 (0.19)**	**−3.44**
Sex	1.35 (0.96)	1.41
BMI	0.04 (0.15)	0.26
SES (average)	−2.88 (2.15)	−1.34
SES (high)	−4.39 (2.23)	−1.97
Urbanicity (average)	−1.80 (1.39)	−1.30
Urbanicity (high)	−1.20 (1.52)	−0.79
*Reactivity slope*	**3.08 (0.47)**	**6.49**
Age*reactivity	**0.07 (0.02)**	**2.92**
Sex*reactivity	**0.26 (0.11)**	**2.39**
BMI*reactivity	**−0.07 (0.02)**	**−4.11**
SES (average)*reactivity	**0.54 (0.25)**	**2.17**
SES (high)*reactivity	**0.83 (0.26)**	**3.20**
Urbanicity (average)*reactivity	**−0.56 (0.16)**	**−3.47**
Urbanicity (high)*reactivity	**−0.87 (0.18)**	**−4.94**
*Recovery slope*	**−1.47 (0.15)**	**−10.02**
Age*recovery	**−0.03 (0.01)**	**−3.86**
Sex*recovery	**−0.12 (0.03)**	**−3.51**
BMI*recovery	**0.02 (0.01)**	**4.65**
SES (average)*recovery	−0.11 (0.08)	−1.47
SES (high)*recovery	**−0.24 (0.08)**	**−3.01**
Urbanicity (average)*recovery	**0.22 (0.05)**	**4.43**
Urbanicity (high)*recovery	**0.36 (0.05)**	**6.58**

**Random effects**	**Variance**	**SD**

Individual	75.02	8.66
Residual	15.43	3.93

*BMI, body mass index; SES, socioeconomic status.

Significant estimates are marked in bold (*T* > 2 or *T* < −2)

### Relation between group and HR responses: Model 2

**Figure 2:**
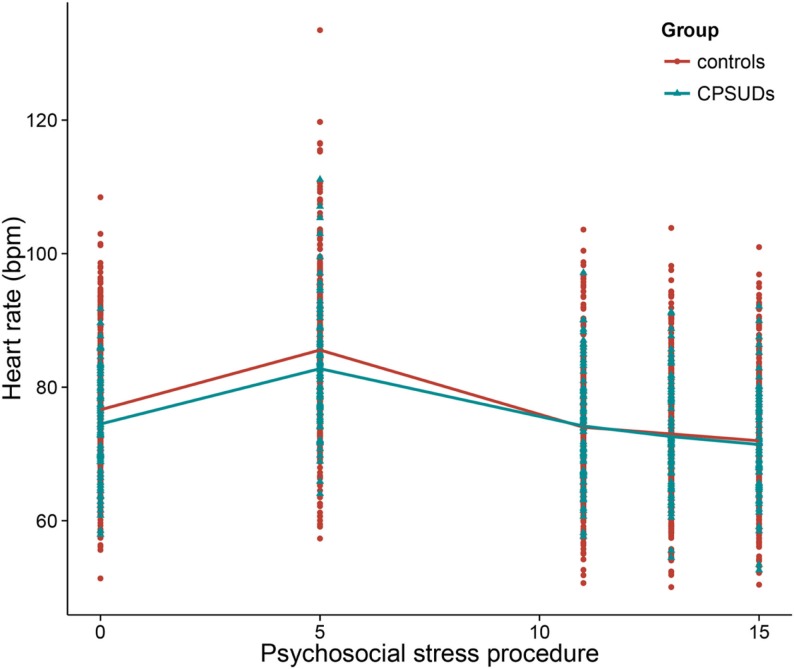
**Heart rate across the psychosocial stress procedure in CPSUDs and controls**. *CPSUDs, children of parents with a substance use disorder; bpm, beats per minute.

In order to address the first aim of the study, which was to examine the relation between group and HR reactivity, we added the variable group (CPSUD versus control) to Model 1, the basic HR response model. We included the main effect (effect of group on the intercept) and interaction effects between group and period (effects of group on the HR reactivity and recovery slopes). This was a significantly improved model above the basic HR response model [χ^2^(3) = 11.31, *p* < 0.05]. CPSUDs did not differ significantly from controls regarding the HR intercept (mean HR at the beginning of the stress procedure) or HR reactivity. Group was significantly related to the HR recovery slope (estimate = 0.13, SE = 0.05, *t* = 2.72) such that CPSUDs showed weaker HR recovery slopes. Figure 2 shows the HR data across the stress procedure in CPSUDs and controls. Model 2 thus consisted of all elements of Model 1 plus the main effect of group and the interactions between group and HR reactivity and HR recovery.

### Potential confounding effects of substance use and externalizing symptoms

The second aim of this study was to examine whether substance use and externalizing symptoms confounded the relation between familial risk group and HR. Because we only observed a significant effect of group on HR recovery, we only tested the potential confounders on this specific relation further.

#### Relation Between Risk Group and Potential Confounders

The first step in testing the second aim of the study was to examine whether CPSUDs differed from controls regarding substance use and externalizing symptoms. The results of these analyses are presented in Table 3. CPSUDs were significantly more likely to have used cannabis ever and to smoke daily, and exhibited more externalizing symptoms than controls. CPSUDs did not differ from controls regarding ever use of alcohol.

**Table 3:** **Parameter estimates of single-level logistic and linear regression models predicting the effect of risk group (predictor) on each potential confounder (outcome)**.

Outcome	Estimate (SE)	*z* or *t*
Alcohol use (never/ever)	0.26 (0.18)	1.44
Cannabis use (never/ever)	**0.94 (0.17)**	**5.60**
Smoking (no/daily smoking)	**0.84 (0.19)**	**4.29**
Externalizing symptoms	**0.45 (0.05)**	**8.53**
Frequency alcohol use	**1.78 (0.45)**	**3.96**
Frequency cannabis use	**0.09 (0.01)**	**9.12**

*Group is coded as 0 = controls and 1 = CPSUDs. Bold values indicate statistical significance at *p* < 0.001. Analyses are controlled for age, sex, body mass index, socioeconomic status, and urbanicity*.

Age and sex were also related to the potential confounders. Older youth were significantly more likely to have used alcohol and cannabis ever and to smoke daily. Girls were significantly more likely to have used alcohol ever and to smoke daily. Sex was not significantly related to cannabis use. Younger youth and boys portrayed significantly more externalizing symptoms.

#### Relation Between Potential Confounders and HR Responses

We then examined whether each of the potential confounders was related to HR responding. We did this by adding each potential confounder (in separate models) to Model 1, the basic HR response model. The results of these analyses are shown in Table 4. Externalizing symptoms was significantly related to HR recovery such that youth who exhibited more externalizing symptoms showed weaker HR recovery slopes. The potential confounders regarding substance use (ever use of alcohol and cannabis and no versus daily smoking) were not related to HR recovery.

**Table 4:** **Parameter estimates of two-level linear regression models predicting the effect of each potential confounder (predictor) on heart rate recovery (outcome)**.

Predictor	Estimate (SE)	*t*
Alcohol use (never/ever)	−0.02 (0.04)	−0.56
Cannabis use (never/ever)	0.01 (0.04)	0.28
Smoking (no/daily smoking)	0.07 (0.05)	1.49
Externalizing symptoms	**0.09 (0.02)**	**4.57**
Frequency alcohol use	0.00 (0.00)	1.15
Frequency cannabis use	0.17 (0.13)	1.30

*Bold values indicate statistical significance at *t* > 2 or *t* < −2. Analyses are controlled for age, sex, body mass index, socioeconomic status, and urbanicity*.

#### Relation Between Group and HR Responses, Controlling for Potential Confounders

In the final step, we tested whether the relation between group and HR responding remained when controlling for the potential confounders. We performed this step only for those potential confounders that were significantly related to both the group variable and HR recovery. Only externalizing symptoms met this criterion. To examine whether number of externalizing symptoms confounded the relation between group and HR recovery, we added the variable externalizing symptoms to Model 2, the model in which group was the main predictor of HR recovery. In this model, the interaction between externalizing and the HR recovery slope was significant (estimate = 0.08, SE = 0.02, *t* = 4.12), and the interaction between group and the HR recovery slope remained significant (estimate = 0.10, SE = 0.05, *t* = 2.12).

### Potential confounding effects of frequency of substance use

The third aim of our study was to examine whether frequency of substance use confounded the relation between group and HR recovery in a subset of youth for whom we had more detailed information on substance use. In this subsample, we first confirmed whether the relation between group and HR recovery was significant, and this was the case (estimate = 0.14, SE = 0.06, *t* = 2.48). Just as in the whole sample, CPSUDs in this subsample did not differ from controls regarding the intercept or the HR reactivity slope.

We then examined whether CPSUDs differed from controls regarding frequency of alcohol and cannabis use. The results of these analyses are given in Table 3. CPSUDs reported drinking alcohol and using cannabis significantly more frequently than controls.

Age and sex also significantly influenced frequency of alcohol and cannabis use such that older youth and boys reported more frequent substance use.

Next, we tested whether frequency of alcohol and cannabis use was significantly related to HR recovery. The results of these analyses are portrayed in Table 4. Neither frequency of alcohol use nor frequency of cannabis use was significantly related to HR recovery. Because of this, we did not test further whether these factors were potential confounders of the relation between group and HR recovery.

## Discussion

Children of parents with a substance use disorder are at increased risk for developing SUDs themselves later in life. Endophenotypes, such as physiological stress reactivity, may help to clarify this familial risk for SUDs. In examining HR across the psychosocial stress procedure, our findings indicated that CPSUDs showed a blunted HR recovery in comparison to control youth from the general population. This is in line with previous findings in adult CPSUDs during a similar stress procedure (21), though in contrast to research in adult and adolescent males in which heightened HR reactivity was found in response to unexpected shock (20) and a mental arithmetic task (19). The present study thus showed that ANS hypo-arousal is more prevalent in youth who have a familial history of SUDs than control youth. One of the criteria for the identification of endophenotypes is that it must be more prevalent in unaffected family members of those diagnosed with the disorder than those from the general population (42, 43). By providing empirical support for this specific criterion, the present study contributes to evidence of ANS hypo-arousal as a potential endophenotype for SUDs.

It has been widely reported that CPSUDs have a greater tendency to engage in risky substance use and also report more externalizing symptoms than their peers [e.g., Ref. (2, 5, 7, 22)]. Considering that risky substance use (23) and externalizing symptoms (24) have both been associated with a blunted HR response, we examined whether substance use and number of externalizing symptoms confounded our finding of a blunted HR recovery in CPSUDs compared to youth from the general population. We found that familial risk group was significantly related to five of the six potential confounders: CPSUDs reported drinking alcohol more frequently, were more likely to smoke daily, were more likely to have used cannabis ever and more frequently, and portrayed more externalizing symptoms. Of the potential confounders, only externalizing symptoms were significantly related to HR recovery. We then included this factor in the model that tested the relation between familial risk group and HR recovery, and this relation remained significant while controlling for externalizing symptoms. This suggests that portraying more externalizing symptoms does not confound the relation between familial risk group and HR recovery. In other words, a familial history of SUDs seems to be related to both blunted HR recovery and an increased risk for portraying externalizing symptoms, independently of one another.

In the present study, CPSUDs did not report being more likely to use alcohol (ever use). In youth, alcohol use (as opposed to alcohol use disorders) may be more normative compared to tobacco or cannabis use. Studies in other samples have also suggested that alcohol use may be less deviant than tobacco or cannabis use in adolescents (44). Quite possibly, this underlies the absence of a relation between familial risk for SUDs and ever alcohol use in the present study. CPSUDs did report drinking alcohol significantly more frequently.

Intriguingly, we observed significant differences in CPSUDs compared to controls for HR *recovery*, but not HR *reactivity*. Mean HR during the recovery periods was lower on average, across groups compared to mean HR during baseline. This was expected and most likely due to anticipatory stress prior to the stressful tasks [e.g., Ref. (45, 46)]. When confronted with a stressor, the adaptive bodily response in humans is immediate and strong activation of the ANS. Following the stressor, it is equally important that this system is deactivated, allowing the body to return to homeostasis. This immediate and strong activation and deactivation should be reflected in an increase in HR followed by a decrease in HR when the stressor has subsided. Alterations from this pattern suggest ANS dysregulation (10). Previous research generally found a blunted HR recovery to indicate health risks [e.g., Ref. (47)] and poor regulation of affect and behavior (48). A reasonable interpretation of the present findings may then be that blunted HR recovery, like blunted HR reactivity, is indicative of a less optimally functioning stress response system. Furthermore, blunted ANS responding has been related to behavioral and emotional problems both in youth from the general population [e.g., Ref. (23, 24, 49, 50)] and in CPSUDs (21, 48). This is consistent with suggestions that physiological stress response dysregulation in youth may signal vulnerability to psychopathology (51). An interesting hypothesis would be to examine whether blunted ANS responding mediates the relation between familial risk for SUDs and behavioral and emotional problems in youth, using longitudinal data. The present study contributes to an empirically based rationale for investigating this.

This study must be considered in light of the following. We were unable to control for pubertal stage as this information was not available for the entire sample of controls. Second, information on frequency of tobacco use was not available. Third, in our control sample, of the 526 youth (11–20 years), only 8 parents had been diagnosed with an SUD (lifetime prevalence). This prevalence rate is substantially lower than is usually reported in general population samples, thus it may be possible that our control sample is biased such that families with problems (such as SUDs) were perhaps less likely to be willing to participate in the study. This does not, however, undermine the generalizability of the study as we excluded youth whose parents had been diagnosed with a SUD. Fourth, we allowed more than one child per family to participate in the study in the CPSUD group. This was done in order to increase the participation rate in that group, as recruitment was challenging. It is not likely that this influenced our findings because we examined in our analyses whether it was necessary to control for family resemblance by including an extra level in the models, and this was not the case.

## Conclusion

In general, our findings suggest a blunted HR recovery from stress in CPSUDs. CPSUDs reported being significantly more likely to have ever used cannabis, to use tobacco daily, to drink alcohol and use cannabis more frequently and portrayed significantly more externalizing symptoms. None of these factors confounded the observed relation between familial risk for SUDs and blunted HR recovery. Our findings contribute to the accumulating evidence for ANS dysregulation as a potential endophenotype for SUDs.

## Author Contributions

AH and IA designed the study. AE, BE, and TK managed the data collection. BE performed the literature search, statistical analyses, and wrote the first draft of the manuscript. AH and KG assisted with the interpretation of the findings. KG contributed significantly to the revision of the manuscript. All authors have critically reviewed and approved the final manuscript.

## Conflict of Interest Statement

The authors declare that the research was conducted in the absence of any commercial or financial relationships that could be construed as a potential conflict of interest.

## Abbreviations

ANS, autonomic nervous system; BMI, body mass index; CBCL, Child Behavior Checklist; CIDI, Composite International Diagnostic Interview; CPSUD, children of parents with a substance use disorder; DSM-IV, diagnostic statistical manual – fourth revision; ECG, electrocardiogram; HR, heart rate; IBI, inter-beat interval; PNS, parasympathetic nervous system; RM-ANOVA, repeated measures analysis of variance; SES, socioeconomic status; SNS, sympathetic nervous system; SUD, substance use disorder; SUQ, Substance Use Questionnaire.
